# Direct Interaction of CD40 on Tumor Cells with CD40L on T Cells Increases the Proliferation of Tumor Cells by Enhancing TGF-β Production and Th17 Differentiation

**DOI:** 10.1371/journal.pone.0125742

**Published:** 2015-05-18

**Authors:** Hyemin Kim, Yejin Kim, Seyeon Bae, Joo Myoung Kong, Jiwon Choi, Mirim Jang, Jiyea Choi, Jun-man Hong, Young-il Hwang, Jae Seung Kang, Wang Jae Lee

**Affiliations:** 1 Laboratory of Vitamin C and Antioxidant Immunology, Department of Anatomy, Seoul National University College of Medicine, Seoul, 110–799, Korea; 2 Institute of Allergy and Clinical Immunology, Seoul National University Medical Research Center, Seoul, 110–799, Korea; Wayne State University School of Medicine, UNITED STATES

## Abstract

It has recently been reported that the CD40-CD40 ligand (CD40L) interaction is important in Th17 development. In addition, transforming growth factor—beta (TGF-β) promotes tumorigenesis as an immunosuppressive cytokine and is crucial in the development of Th17 cells. This study investigated the role of CD40 in breast cancer cells and its role in immunosuppressive function and tumor progression. CD40 was highly expressed in the breast cancer cell line MDA-MB231, and its stimulation with CD40 antibodies caused the up-regulation of TGF-β. Direct CD40-CD40L interaction between MDA-MB231 cells and activated T cells also increased TGF-β production and induced the production of IL-17, which accelerated the proliferation of MDA-MB231 cells through the activation of STAT3. Taken together, the direct CD40-CD40L interaction of breast tumor cells and activated T cells increases TGF-β production and the differentiation of Th17 cells, which promotes the proliferation of breast cancer cells.

## Introduction

CD40 is a 50 kDa type I transmembrane protein that belongs to the tumor necrosis factor (TNF)-receptor superfamily [1]. It is physiologically expressed in various cell types including dendritic cells, B cells and monocytes/macrophages [2]. Especially, CD40 in B cells has a critical role in the development and proliferation of B cells by enhancing interleukin (IL)-2, IL-4, and IL-5 and the production of other chemokines. [3–6]. CD40 also induces the differentiation of B cells into antibody-secreting plasma cells [7, 8]. The ligand for CD40 is CD154 (CD40L) which is predominantly expressed in activated T cells and has a crucial role in the regulation of B cell proliferation [9, 10]. Recently, it was reported that CD40-CD40L cross-talk is important in Th17 development [11]. In addition to B cells, CD40 is also expressed in several kinds of tumor cells, such as breast cancer, ovarian cancer, colon cancer, and melanoma [10, 12]; however, its specific roles are still largely unknown.

Transforming growth factor-beta (TGF-β) is a pleiotropic cytokine that controls multiple cellular responses including the induction of cell growth inhibition, differentiation, cellular senescence, wound healing and apoptosis [13]. TGF-β cannot bind to its receptors in its latent form which is regulated by latency-associated protein and latent- TGF-β binding protein [14]. In vitro, these factors can be inactivated by extreme pH or heat, or by several proteases [15]. However, *in vivo* mechanisms for the activation of TGF-β are less clear; however, several models have been proposed including proteolysis by transglutaminase, conformational change of latency-associated proteins through physical interaction with thrombospondin, and modulation by αvβ6 integrins in epithelial cells [16–18]. TGF-β has dual roles in the progression and metastasis of cancer [19]. In human cancers, TGF-β promotes tumorigenesis through both decreased TGF-β signaling during early tumorigenesis and increased TGF-β signaling in advanced, progressive disease [13, 20]. TGF-β is a potent suppressor of proliferation in normal epithelial cells, notably breast; however, it converts to a promoter during cancer development [21]. In particular, TGF-β signaling has important roles during breast cancer progression and metastasis in various mouse models [19, 22, 23], and the level of TGF-β was increased in cancer patients [24, 25]. TGF-β has a role in the differentiation of CD4^+^CD25^+^ regulatory T cells which potently suppress both *in vitro* and *in vivo* effector T cell function and maintain Foxp3 expression [26–28], and it is also essential in the induction of Th17 cells [29, 30].

This study investigated the role of CD40 in the production of TGF-β in breast cancer cells, and the results show that the production of TGF-β induced by the CD40-CD40L interaction, results in the enhanced immunosuppressive function of breast cancer cells and could thereby contribute to tumor progression.

## Materials and Methods

### Cells

The human breast cancer cell lines, MDA-MB231 and HS-578T were purchased from American Type Culture Collection (Manassas, VA, USA). Cells were maintained in continuous log phase of growth at 37°C in a humidified atmosphere containing 5% CO_2_ with RPMI 1640 medium supplemented with 2 mM L-glutamine, 100 units/ml penicillin, 100 μg/ml streptomycin (Welgene, Daegu, Korea), and 10% heat-inactivated fetal bovine serum (FBS, Hyclone, Utah, USA).

### Isolation of T cells from human peripheral blood

Heparinized peripheral blood was collected from healthy volunteers under protocol approved by an Institutional Review Board (IRB) of Seoul National University Hospital (SNUH) (IRB#:0902-022-271). Human T cells were enriched from peripheral blood by using RosetteSep (Stem Cell Technologies, Vancouver, Canada). Briefly, 40 ml of blood obtained from normal healthy volunteer was mixed with 2 ml of RosetteSep cocktail consisted of mouse IgG1 antibodies to human lineage antigens (CD16, CD19, CD36 and CD56) and incubated at room temperature for 30 min with gentle mixing. After dilution with an equal volume of phosphate buffered saline (PBS), T cells were isolated by density gradient centrifugation using pre-warmed Ficoll-Paque (GE healthcare lifesciences, Uppsala, Sweden) at 600 g for 20 min. The interface was harvested, centrifuged at 2,000 rpm for 10 min, and then pellet was suspended to RPMI 1640 medium contained 10% FBS. Otherwise, peripheral blood was mixed with an equal volume of PBS, and loaded onto pre-warmed Ficoll-Paque. After centrifuging at 600 g for 20 min, a buffy coat containing PBMC was harvested and washed with PBS twice. The red blood cells (RBCs) were lysed with RBC lysis buffer (Sigma, St. Louis, MO, USA) in a 37°C water bath for 5 min with shaking, and the mononuclear cells were washed and counted. Human T cells among the isolated mononuclear cells were separated by using the Pan T Cell Isolation Kit (Miltenyi Biotec, Germany) with autoMACS Pro Separator (Miltenyi Biotec, Germany) according to the manufacturers' instruction. In brief, determined cells were suspended with buffer and mixed with biotin-antibody cocktail (10 μl/10^7^ cells) for 5 min at 4°C. After washing, cells were mixed with anti-biotin microbeads (20 μl/10^7^ cells) for 10 min at 4°C. Washed cells were applied to the autoMACS separator, and negatively selected T cells were counted. We confirmed more than 95% of purified T cells were CD3^+^ cells by flow cytometry analysis, after staining with PE-conjugated anti-CD3 antibody (eBioscience, San Diego, CA, USA).

### Activation of T cells

CD4 expression on activated T cells was reduced by stimulation with phorbol 12- myristate 13-acetate (PMA)/ionomycin reduces, but not by phytohemagglutinin (PHA) [31, 32]. However, PHA alone cannot effectively induces CD40L, but in combination with PMA showed CD40L expression comparable to those seen with a combination of CD3 mAb and PMA [33]. Purified T cells (2×10^6^/ml) were activated by of 5 μg/ml of PHA (Life Technologies, Grand Island, NY) for 69 hrs, and then activated with 10 ng/ml of PMA (Sigma, St.Louis, MO, USA) and 1 μg/ml of ionomycin for another 3 hrs. Activated T cells were analyzed by flow cytometry after staining with FITC-conjugated anti-CD69 or CD25 antibodies (BD Pharmingen, San Diego, CA, USA).

### Flow cytometry analysis

MDA-MB231 cells were stained with PE-conjugated anti-human CD40 antibody (BD Pharmingen, San Diego, CA, USA), and activated T cells were stained with FITC-conjugated anti-CD25 antibody or PE-conjugated anti-CD40L antibody (BD Pharmingen, San Diego, CA, USA) for 30 min on ice. After washing with buffer containing 0.5% bovine serum albumin (BSA) in PBS, stained cells were analyzed by FACS Calibur (BD Bioscience, San Jose, CA, USA). To determine the Th17 differentiation by the ligation of CD40L on activated T cells with CD40 expressing MDA-MB231 cells or anti-CD40L agonistic antibody (2 μg/ml), intracellular IL-17 staining with Alexa Fluor 647-conjugated anti-IL-17 antibody and surface CD4 staining with FITC-conjugated anti-CD4 antibody was performed by BD Cytofix/Cytoperm Fixation/Permeabilization Solution Kit (BD Pharmingen, San Diego, CA, USA), and RORγt staining with PE-conjugated anti- RORγt antibody was performed by using a transcription factor staining buffer set (eBioscience, San Diego, CA, USA) according to the manufacturer’s instructions. Flow cytometry was performed with FACS Calibur, and analyzed with FlowJo software (Ashland, OR, USA).

### [^3^H]-Thymidine incorporation assay

MDA-MB231 cells growing at logarithmic growth phase were trypsinized and resuspended in growth media at 2.5×10^4^ cells/ml. A hundred microliter of the cell suspension was dispensed onto a 96-well plate. Quadruplicate samples were incubated in the presence or absence of agonistic anti-CD40 antibody (2, 4, 8, and 16 μg/ml) for 24 hrs. After addition of 1 μCi/mL of [^3^H]-thymidine to each well, 96-well plate was further incubated for 18 hrs at 37°C in a 5% CO_2_ atmosphere. Then, cells were harvested onto glass fiber filters using a cell harvester (Inotech Biosystems International, Dietikon, Switzerland). Glass fiber filters were dried and sealed into polythene bags with scintillation fluid (Betaplate Scint; PerkinElmer, Boston, MA, USA). The incorporation of [^3^H]-thymidine counted on a MicroBeta Trilux1450 (PerkinElmer, Boston, MA, USA).

### Co-culture of MDA-MD231 cells and T cells

To investigate the requirement of interaction between CD40 and CD40L for IL-1β, IL-6, IL-21 and TGF-β production from MDA-MB231 cells and IL-17 differentiation from activated T cells, MDA-MB231 cells (1.5 x 10^5^/well) were incubated with activated T cells (AT) and resting T cells (RT) in the same well on 6-well plate for 24 hrs at the ratio of 1:5, 1:10 and 1:20. And it was confirmed by the interference of interaction between CD40 and CD40L with anti-CD40 neutralizing antibody (2 μg/ml). It was also confirmed by the co-culture of MDA-MB231 cells and activated T cells by using of a Transwell culture system (Corning Life Sciences, Tewksbury, MA, USA). Briefly, MDA-MB231cells (1.5×10^5^/well) were cultured in the lower chamber (25 mm) of the Transwell system and activated T cells (7.5×10^5^/well) were loaded on the upper chamber with a polycarbonate membrane bottom (0.4 μm pore size). Culture supernatants were harvested, centrifuged, and frozen in aliquots for further experiments.

### Measurement of cytokine levels by ELISA

MDA-MB231 cells were stimulated with anti-CD40 agonistic antibody (2 μg/ml) or isotype (2 μg/ml). And then culture supernatants were harvested and stored at -70°C until use. The concentration of TGF-β (Biosource International, Inc. Camarillo, CA, USA), IL-1β, IL-6 (R&D system, Minneapolis, MN, USA) and IL-21 (eBioscience, San Diego, CA, USA) was measured with commercially available Enzyme Linked Immunosorbent assay (ELISA) kits, according to the manufacturers’ instruction. The final concentration of cytokines was normalized with cell numbers.

### Reverse Transcriptase-Polymerase Chain Reaction (RT-PCR) and Real-time PCR

MDA-MB231 cells were seeded on a 24-well plate (1.5 x 10^5^/well) and incubated overnight. Then, anti-CD40 agonistic antibody (2 μg/ml) and isotype antibody (Sigma, St. Louis, MO, USA) were treated to MDA-MB231 cells for 3, 6, 9 and 12 hrs. Total RNA was extracted from CD40-stimulated MDA-MB-231 cells by using Trizol (Invitrogen life technologies, Carlsbad, CA, USA), according to the manufacturers’ instruction, and RNAs were quantified with NanoDrop (Thermo scientific, Wilmington, DE, USA). Total RNA (1 μg) was transcribed to cDNA by avian myeloblastosis virus (AMV) reverse transcriptase (Promega, Madison, WI, USA). For conventional RT-PCR, cDNAs were amplified with flowing primers. TGF-β: Forward, 5′-GGG ACT ATC CAC CTG CAA GA-3′ and Reverse, 5′-CCT CCT TGG CGT AGT AGT CG-3′; β-actin: Forward, 5′-TCC TTA ATG TCA CGC ACG A-3′ and Reverse, 5′-GTG GGG CGC CCA GGC ACC A-3′. PCR products were separated on 1.5% agarose gel, stained with ethidium bromide and visualized under UV light. Quantitative Real-time RT-PCR analysis was performed by using SYBR Green Master mix (Fermentas, Hudson, NH, USA), and primers were used as followed. TGF-β: Forward, 5′- GGC GAT ACC TCA GCA ACC G-3′ and Reverse, 5′-CTA AGG CGA AAG CCC TCA AT-3′, GAPDH: GCC ACC CAG AAG ACT GTG GA-3′ and Reverse, 5’-CAG TGA GCT TCC CGT TCA GC-3’. Amplification conditions were: 95°C for 10 min, followed by 45 cycles of 95°C for 30s, 57°C for 30s and 72°C for 15s, and followed by an extension at 72°C for 5 min. For assessing the mRNA expression level, the Ct value for TGF-β was subtracted from the Ct value of GAPDH to yield a ΔCt value. The average ΔCt was calculated for the control group and subtracted from the ΔCt of all other samples (including the control group). This resulted in a ΔCt value for all samples used to calculate the fold-induction of mRNA expression of target gene using the formula 2-ΔCt.

### Western blot analysis

MDA-MB231 cells (1.5 x 10^5^/well) were incubated with recombinant IL-17 or co-culture supernatants of MDA-MB231 cells and activated T cells for 15, 30, 60 min on 6-well plate. After washing with PBS, cells were homogenized with lysis buffer and total proteins were quantified by bicinchoninic acid (BCA) assay. Equal amounts of protein were resolved over 10% polyacrylamide gel and transferred to a nitrocellulose membrane. Followed blocking with 5% nonfat milk, membranes were incubated with anti-phospho-STAT3 antibody (1:200), anti-STAT3 antibody (1:200) (Cell signaling, Danvers, MA, USA) or β-actin (1:4000, Sigma, St. Louis, MO, USA) at 4°C for overnight. After incubating with horseradish peroxidase-conjugated anti-rabbit or anti-mouse IgG (1:1000, Cell signaling, Danvers, MA, USA), the immunoreactive proteins were visualized with the ECL detection system (Amersham Biosciences Corp., Piscataway, NJ, USA).

### CD40 siRNA transfection

CD40 siRNA and control siRNA were purchased from Santa Cruz Biotechnology (Palo alto, CA, USA). Cells in exponential phase of growth were plated in 6-well plates at 5 x 10^5^ cells/well, grown for 24 hrs and then transfected with 20 nM of siRNA using oligofectamine and OPTI MEM I-reduced serum medium (Invitrogen Life Technologies, Carlsbad, CA, USA), according to the manufacturer’s protocol. The concentrations of siRNA were chosen based on dose-response studies. Transfection efficiency was examined by flow cytometry after staining with PE-conjugated anti-CD40 antibody at 72 hrs after transfection. Control cells were transfected with control siRNA with oligofectamine and serum-reduced medium (mock).

### Statistics

Data were expressed as mean ± S.D. of each group in independent experiments. For comparison of three or more groups, data were analyzed by t-test or one-way analysis of variance (ANOVA) followed by Newman-Keuls multiple comparison tests. A value of P < 0.05 was considered statistically significant. Statistical tests were carried out using GraphPad InStat (GraphPad Software, San Diego, CA, USA).

## Results

### CD40 stimulation up-regulates TGF-β production in the malignant breast cancer cell line, MDA-MB231

**Fig 1:**
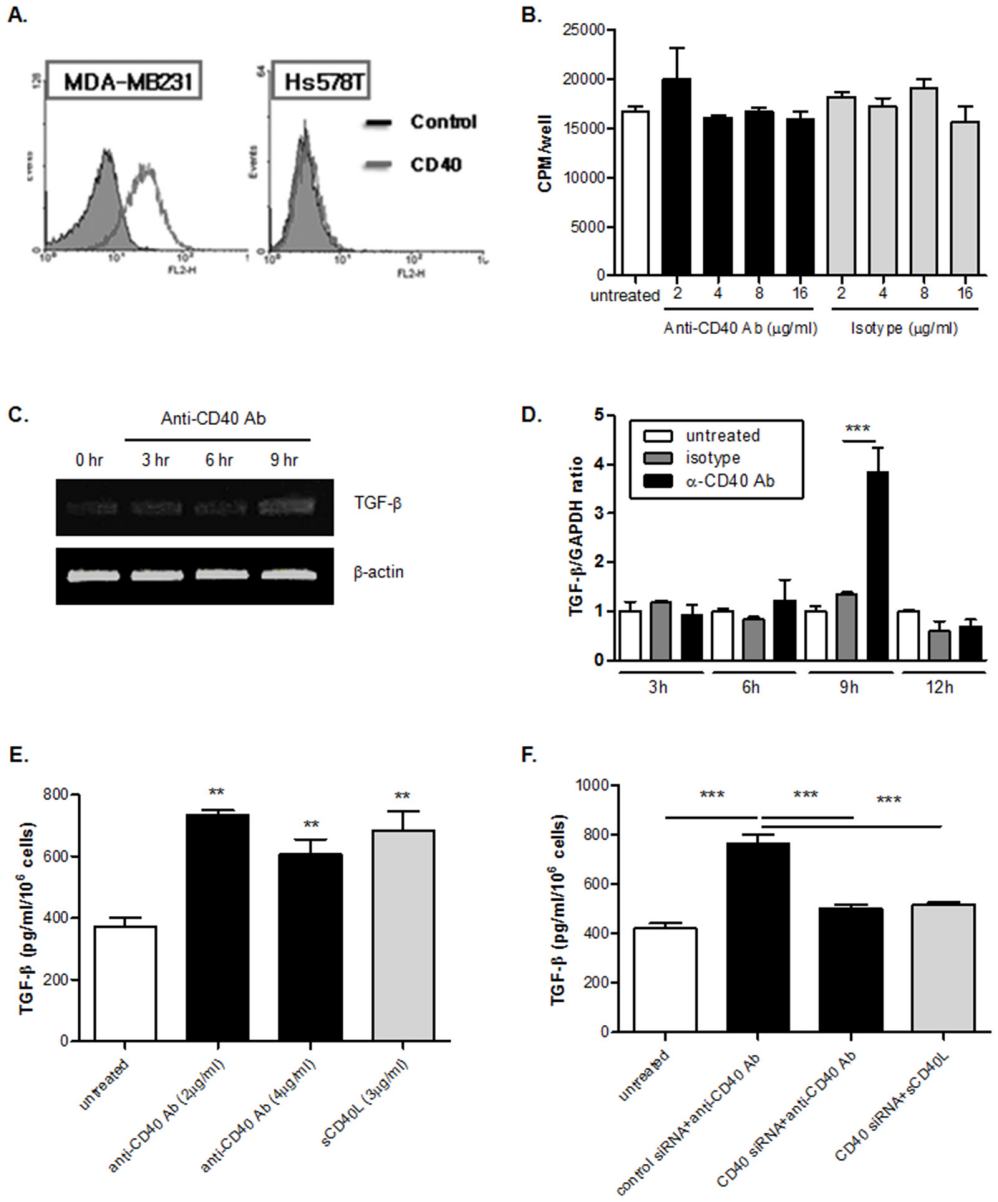
TGF-β production is up-regulated by CD40 stimulation in a breast cancer cell line, MDA-MB231. (A) The breast cancer cell lines, MDA-MB231 and Hs578T were collected at continuous log phase of growth. The expression of CD40 was examined by staining with PE-conjugated anti-human CD40 antibody (1 μg/ml) as described in *Materials and Methods*. Result is the representative of three independent experiments. (B) MDA-MB231 cells (2.5 x 10^3^/well) were stimulated with the various concentrations of anti-CD40 agonistic antibody (2, 4, 8, and 16 μg/ml) and the same amount of isotype antibody for 1 hr on 96-well plate, and then cells were cultured for 24 hrs. After the addition of 1 μCi/mL of [^3^H]-thymidine, cells were culture for another 18 hrs. And then, the proliferation of cells by CD40 stimulation was measured as described in *Materials and Methods*. Data represents mean ± S.D. The assay was performed in quadruplicate and result is the representative of three independent experiments. There was no statistical significance among groups. (C and D) The expression of TGF-β mRNA transcript in MDA-MB231 cells by CD40 stimulation was investigated with semi-quantitative RT-PCR (C) and quantitative real-time PCR (D). Cells (1.5 x 10^5^/well) were stimulated 2 μg/ml of anti-CD40 agonistic antibody for 3, 6, 9 and 12 hrs on 6-well plate. Cells were harvested and total RNA was extracted with Trizol, according to manufacturer’s instruction. After quantification, 1μg of RNA was converted to cDNA by reverse transcriptase. And then RT-PCR and real-time PCR were performed with specific primers for TGF-β as described in *Materials and Methods*. β-actin or GAPDH was used as a loading control. ***p < 0.001. (E) CD40 on MDA-MB231 cells (1.5 x 10^5^/well) were stimulated with anti-CD40 agonistic antibody (2 and 4 μg/ml) and soluble CD40L (3 μg/ml) for 1 hr, and then cells were cultured for 24 hrs on 6-well plate. The amount of TGF-β in the culture supernatant was measured by ELISA, according to manufacturer’s instruction. Data represents mean ± S.D. Result is the representative of three independent experiments and each experiment was performed in triplicate. **p < 0.01 vs. untreated control. (F) Cells (1.5 x 10^5^/well) were seeded on 6-well plate and then transfected with 20 nM of CD40 siRNA and control siRNA in the mixture of serum free media and oligofectamine as described in *Materials and Methods*. After 72 hrs, the down-regulation of CD40 expression on MDA-MB231 cells by CD40 siRNA transfection was confirmed by flow cytometry analysis. And then, cells (1.5 x 10^5^/well) were stimulated with anti-CD40 agonistic antibody (2 μg/ml) and soluble CD40L (3 μg/ml) for 1 hr, and then cells were cultured for 24 hrs on 6-well plate. And then, the amount of TGF-β in the culture supernatant was measured by ELISA. Data represents mean ± S.D. Result is the representative of three independent experiments and each experiment was performed in triplicate. ***p < 0.001 vs. untreated control.

First, the expression of CD40 was examined in the human breast cancer cell lines MDA-MB231 and Hs578T. MDA-MB231 cell is known to be more malignant than Hs578T cell. As shown in Fig 1A, CD40 is highly expressed on the surface of the MDA-MB231 cells; however, Hs578T cells hardly express CD40 on the surface. Because CD40 stimulation is known as a critical factor in the proliferation and differentiation of B cells [33], whether the proliferation of MDA-MB231 cells is also increased by stimulation of CD40 with anti-CD40 antibody was also investigated. The stimulation of CD40 in MDA-MB231 did not affect the proliferation of the MDA-MB231 cells (Fig 1B). Thus, we focused on the effect of CD40 stimulation in the production of immune modulatory molecules from tumor cells. TGF-β and IL-10 are the most well-known immunosuppressive molecules produced by tumor cells [34]. The transcriptional expression of TGF-β was determined by RT-PCR, and TGF-β mRNA transcripts were increased by CD40 stimulation with the anti-CD40 antibody (Fig 1C). This result was also confirmed by real-time PCR (Fig 1D). Nine hours after stimulating CD40 in MDA-MB231 cells, TGF-β mRNA transcripts were increased and then decreased by the twelfth hour. Next, the production of TGF-β from MDA-MB231 cells was measured after treatment with anti-CD40 antibody or soluble CD40 ligand (sCD40L) (Fig 1E). MDA-MB231 cells secreted more TGF-β in response to CD40 stimulation. To confirm the TGF-β production from MDA-MB231 cells by CD40 stimulation on the surface, TGF-β production was measured from CD40 siRNA transfected MDA-MB231 cells. A decrease in CD40 expression on the surface of MDA-MB231 cells by CD40 siRNA transfection was confirmed (S1 Fig). As a result, TGF-β production was decreased from the CD40 siRNA transfected MDA-MB231 cells by CD40 stimulation (Fig 1F). Therefore, it seems that CD40 in MDA-MB231 triggers TGF-β production when it is stimulated with anti-CD40 Ab or CD40 ligand (CD40L).

### A direct CD40-CD40L interaction between MDA-MB231 cells and activated T cells increases TGF-β production

**Fig 2:**
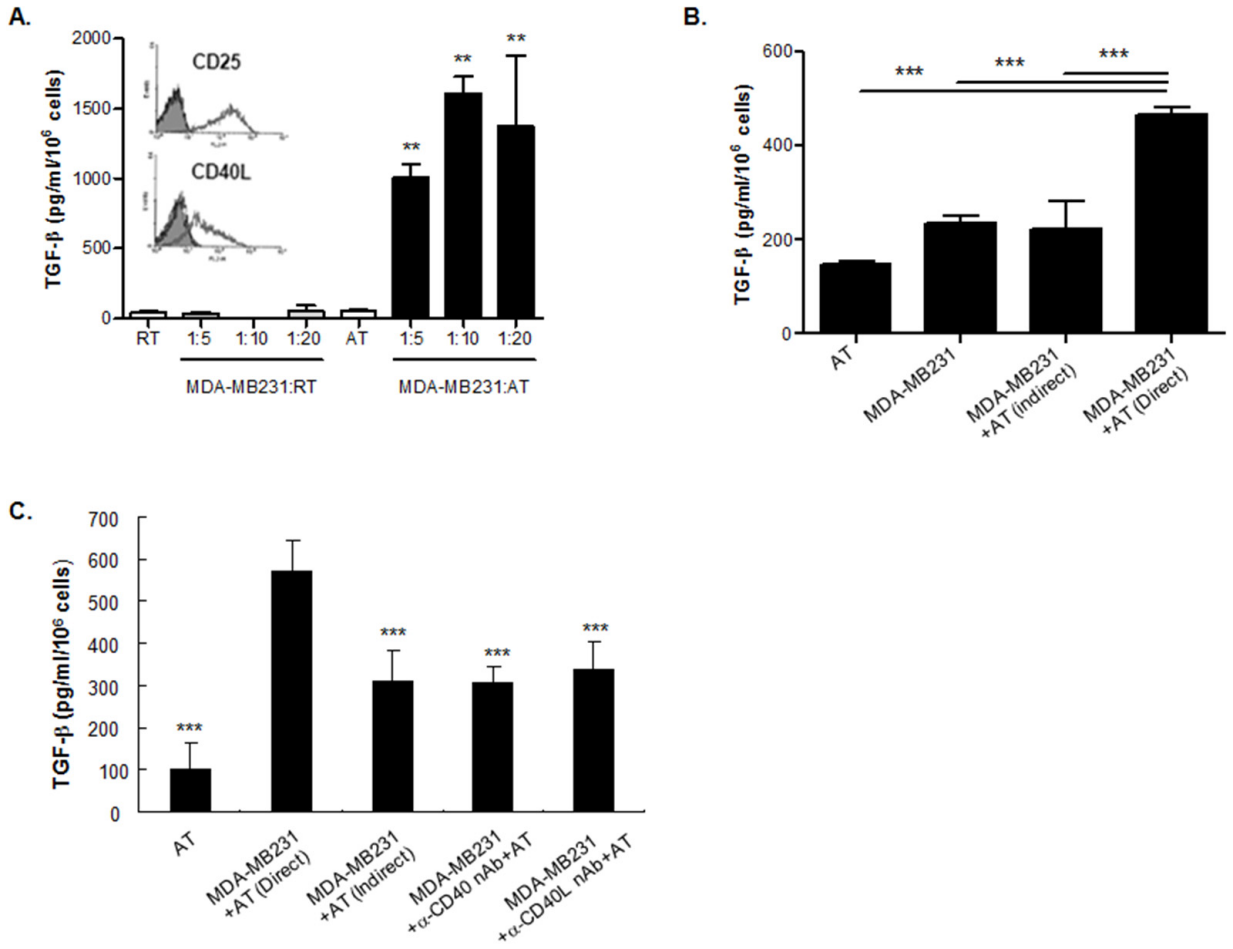
Direct interaction between CD40 on MDA-MB231 cells and CD40L on activated T cells increases TGF-β production. (A) Human T cells (2 x 10^6^/ml) purified from peripheral blood mononuclear cells (PBMCs) were stimulated with 10 ng/ml PMA + 1 μg/ml ionomycin in 0.5% phytohemmaglutinin (PHA)-contained media for 16 hrs on 6-well plate. The expression of CD25 and CD40L on the activated T cells was confirmed by staining with FITC-conjugated anti-human CD25 antibody (1 μg/ml) and PE-conjugated anti-human CD40L antibody (1 μg/ml), respectively as described in *Materials and Methods*. After MDA-MB231 cells (1.5 x 10^5^/well) were incubated with activated T cells (AT) and resting T cells (RT) in the same well on 6-well plate for 24 hrs at the ratio of 1:5, 1:10 and 1:20, the amount of TGF-β in the culture supernatant was measured by ELISA, according to manufacturer’s instruction. Data represents mean ± S.D. Result is the representative of three independent experiments and each experiment was performed in triplicate. **p < 0.01 vs. activated T cells. (B) MDA-MB231 cells were directly co-cultured with activated T cells in the same well on 6-well plate at the ratio of 1:5 for 24 hrs. Also, MDA-MB231 cells and activated T cells were co-cultured in a Transwell system to inhibit direct contact; MDA-MB231 cells were in lower chamber and T cells were in the upper chamber on 6-well plate at the ratio of 1:5 for 24 hrs. The amounts of TGF-β in the culture supernatants from both culture systems were measured by ELISA, according to manufacturer’s instruction. Data represents mean ± S.D. Result is the representative of three independent experiments and each experiment was performed in triplicate. ***p < 0.001. (C) MDA-MB231 cells and activated T cells were directly or indirectly co-cultured as shown in Fig 2B. And, MDA-MB231 cells and activated T cells were directly co-cultured in the same well on 6-well plate at the ratio of 1:5 for 24 hrs, after pre-incubation of MDA-MB231 cells (1.5 x 10^5^/well) with anti-CD40 neutralizing antibody (2 μg/ml) or pre-incubation of activated T cells with anti-CD40L neutralizing antibody for 1hr. The amount of TGF-β in the culture supernatant was measured by ELISA, according to manufacturer’s instruction. Data represents mean ± S.D. Result is the representative of three independent experiments and each experiment was performed in triplicate. ***p < 0.001 vs. direct co-culture of MDA-MB231 cells and activated T cells.

Next, the effect of activated T cells on TGF-β production was investigated because CD40L is known to be expressed in activated T cells [1]. The expression of CD40L and CD25 in the activated T cells was confirmed by flow cytometry (Fig 2A, insert). Then, MDA-MB231 cells were co-cultured with the activated T cells for 24 hrs, and the TGF-β level in the culture supernatant was examined by ELISA. TGF-β production was remarkably increased after co-culture with activated T cells but not with resting T cells (Fig 2A). However, the production of TGF-β did not increase when the direct contact between the MDA-MB231 cells and the activated T cells were inhibited by the inserts of the Trans-well system (Fig 2B). This result suggests that the production of TGF-β is mediated by direct contacts between MDA-MB231 cells and activated T cells, not by soluble factors. We also performed a neutralizing experiment to clarify that the increased TGF-β production is mediated by the interaction between the CD40 on the MDA-MB231 cells and the CD40L on the activated T cells. TGF-β production was inhibited when the activated T cells were pre-incubated with anti-CD40L neutralizing antibody prior to co-culture with the MDA-MB231 cells. In addition, the production of TGF-β was blocked when MDA-MB231 cells were pre-incubated with anti-CD40 neutralizing antibody (Fig 2C). Therefore, a direct interaction between MDA-MB231 cells and activated T cells induces the production of TGF-β.

### A direct interaction between CD40 on MDA-MB231 cells and CD40L on activated T cells induces Th17 differentiation

**Fig 3:**
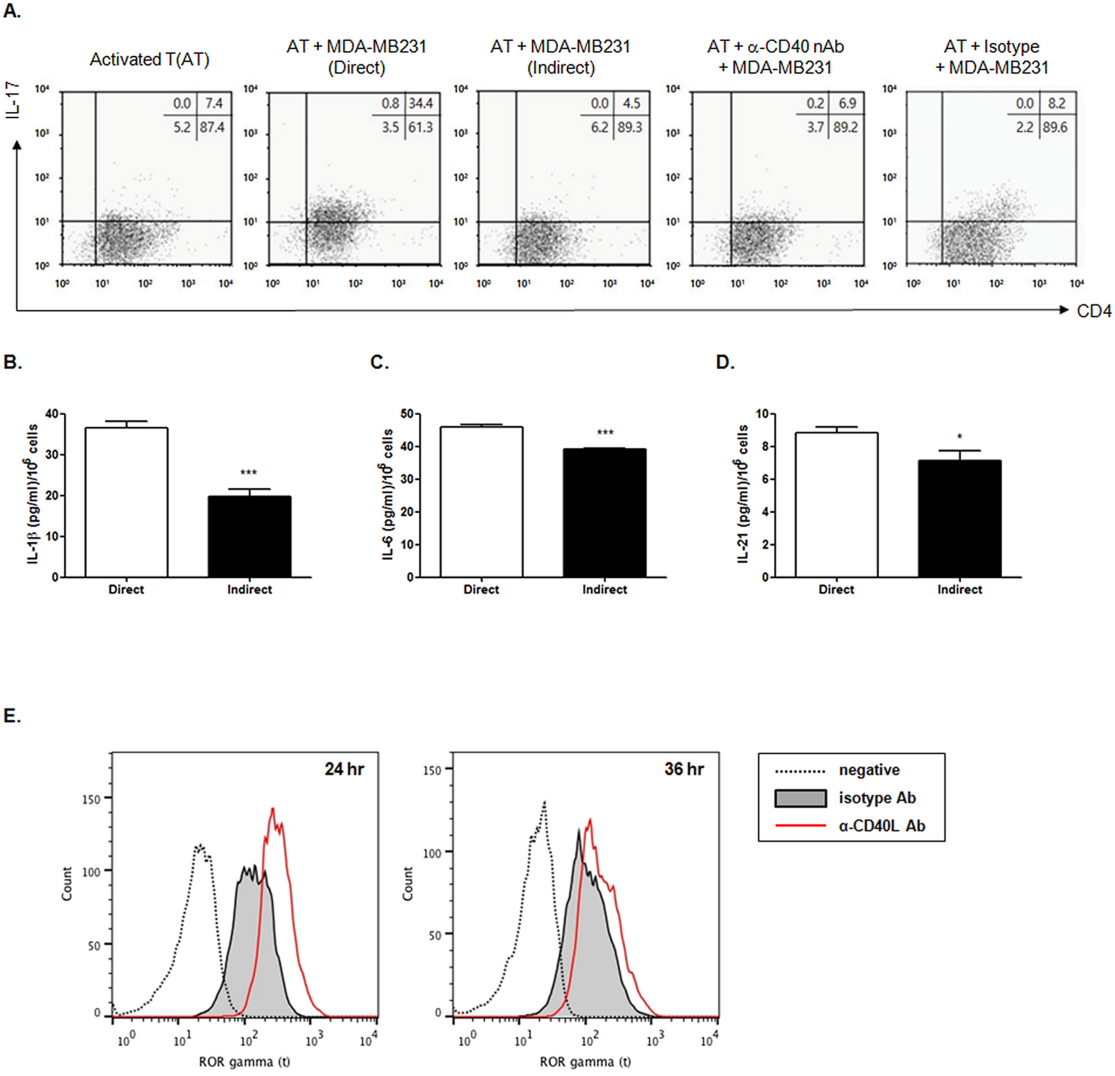
CD40 ligand stimulation on the activated T cells induces Th17 differentiation. (A) Human T cells (2.5x10^6^/ml) purified from human PBMCs were activated as described in *Materials and Methods*. After increase of CD40L expression on activated T cells was confirmed by flow cytometry analysis, they were co-cultured with MDA-MB231 cells in the same well on 6-well plate at the ratio of 5:1 for 24 hrs. Th17 differentiation was confirmed by staining of intracellular IL-17 in CD4^+^ T cells with Alexa Fluor 647-conjugated anti-human IL-17A antibody (1 μg/ml) and FITC-conjugated anti-human CD4 antibody (1 μg/ml). To confirm the role of CD40 on Th17 differentiation, the interaction between CD40 and CD40L was interfered with the addition of anti-CD40 neutralizing antibody (2 μg/ml) for 1 hr. Result is the representative of three independent experiments. (B-D) Culture supernatant from the experiment described in Fig 3A were collected and the amount of IL-1β (B), IL-6 (C) and IL-21 (D) were measured by ELISA, according to manufacturer’s instruction. Data represents mean ± S.D. Result is the representative of three independent experiments and each experiment was performed in triplicate. *p < 0.05. (E) Human T cells (2.5x10^6^/ml) purified from human PBMCs were activated as described in *Materials and Methods*. After increase of CD40L expression on activated T cells was confirmed by flow cytometry analysis, activated T cells were stimulated by anti-CD40L agonistic antibody (1 μg/ml) or isotype (1 μg/ml) for 24 and 36 hrs. The expression of ROR gamma t (RORγt), the orphan nuclear receptor for IL-17 transcription and Th17 differentiation was determined by intracellular flow cytometry analysis, after staining with PE-conjugated anti- RORγt antibody (1 μg/ml) and PE-conjugated Rat IgG (1 μg/ml) as described in *Materials and Methods*. Result is the representative of three independent experiments.

**Fig 4:**
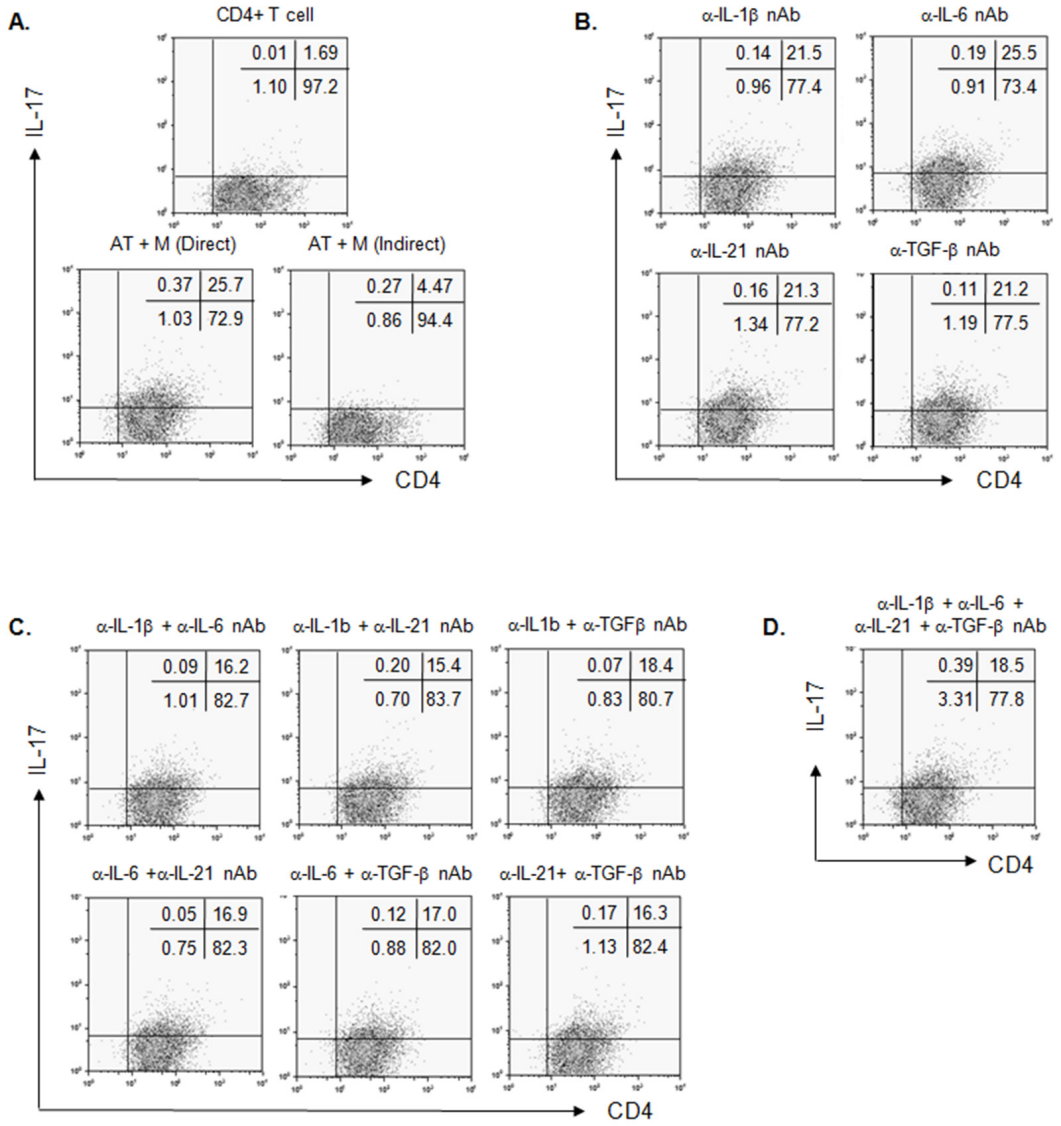
Direct interaction of CD40 and CD40L induces the differentiation of IL-17-producing cells. Human T cells (2.5x10^6^/ml) purified from human PBMCs were activated as described in *Materials and Methods*. (A) After increase of CD40L expression on activated T cells was confirmed by flow cytometry analysis, MDA-MB231 cells and activated T cells were directly or indirectly co-cultured at the ratio of 1:5 for 24 hrs as shown in Fig 2B. (B) MDA-MB231 cells and activated T cells were directly co-cultured at the ratio of 1:5 for 24 hrs in the presence of anti-IL-1β, IL-6, IL-21 and TGF-β neutralizing antibodies (1 μg/ml/each). (C) MDA-MB231 cells and activated T cells were directly co-cultured at the ratio of 1:5 for 24 hrs in the presence of the combination two of each antibody against IL-1β, IL-6, IL-21 and TGF-β (1 μg/ml/each). (D) MDA-MB231 cells and activated T cells were directly co-cultured at the ratio of 1:5 for 24 hrs in the presence of the combination all of antibodies against IL-1β, IL-6, IL-21 and TGF-β (1 μg/ml/each). Th17 differentiation in shown in Fig 4 was confirmed by staining of intracellular IL-17 in CD4^+^ T cells with Alexa Fluor 647-conjugated anti-human IL-17A antibody (1 μg/ml) and FITC-conjugated anti-human CD4 antibody (1 μg/ml). Each result is the representative of three independent experiments.

TGF-β is a critical factor in Th17 cell differentiation [35]. Because a direct interaction between CD40 on MDA-MB231 cells and CD40L on activated T cells increased TGF-β production, IL-17 production in activated T cells was examined. When activated T cells were directly co-cultured with MDA-MB231 cells, there was a large increase in IL-17-producing cells shown in Fig 3A. However, this increase was attenuated by indirect interaction with Transwell or by direct interaction between the activated T cells and MDA-MB231 cells treated with anti-CD40 neutralizing antibodies (Fig 3A). The cytokines IL-1β, IL-6 and IL-21 are also important in Th17 cell differentiation in humans [36]. Thus, the production of cytokines IL-1β, IL-6 and IL-21 secreted into the media was assessed with the direct or indirect co-culture of MDA-MB231 cells and activated T cells. There was an increased level of IL-1β, IL-6 and IL-21 in the direct co-culture compared to the indirect co-culture (Fig 3B–3D). This result suggests that Th17 differentiation is stimulated by cytokines induced by the direct interaction between activated T cells and MDA-MB231 cells. Additionally, the CD40-CD40L interaction has a key role in this process. Retinoic acid receptor-related orphan nuclear receptor gamma t (RORγt), an orphan nuclear receptor, regulates IL-17 transcription and Th17 differentiation [37] Therefore, we investigated whether CD40-CD40L interaction increases RORγt expression in activated T cells. As seen in Fig 3E, RORγt expression is increased when CD40L on activated T cells interacts with the anti-CD40 antibody, regardless of the presence of cytokines for Th17 differentiation such as TGF-β, IL-1β, IL-6, and IL-21. As shown in Fig 4, the increase in IL-17 producing cells through the direct interaction of the MDA-MB231 cells and the activated T cells was not completely but partially attenuated by treatment with one or more neutralizing antibodies against IL-1β, IL-6, IL-21 or TGF-β (Fig 4B and 4C). In addition, Th17 differentiation was not completely prevented by antibodies against TGF-β, IL-1β, IL-6, and IL-21 (Fig 4D). It means that Th17 differentiation could be only induced by CD40-CD40L interaction.

### IL-17 induces proliferation of MDA-MB231 cells by activation of STAT3

**Fig 5:**
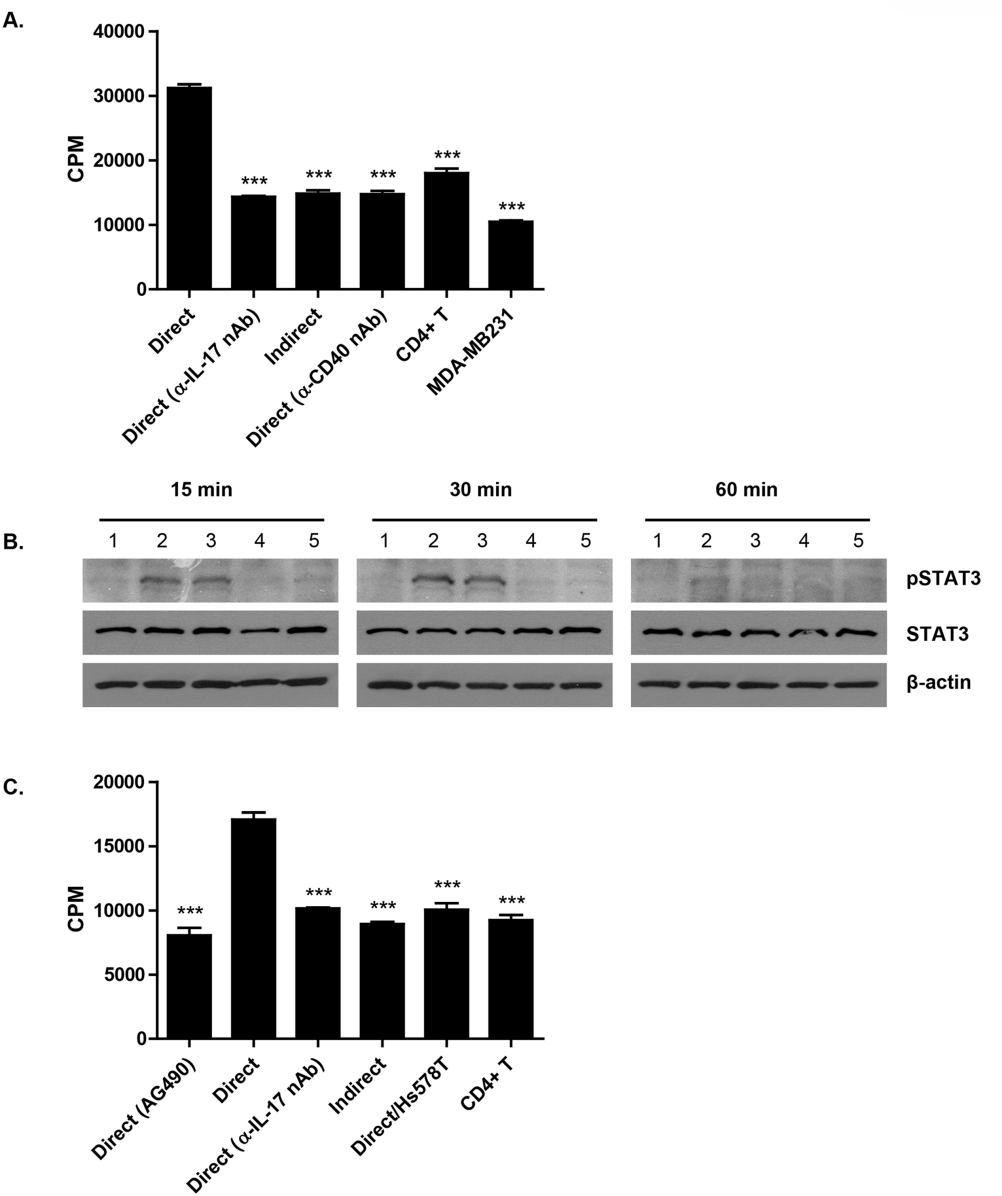
IL-17 production via direct interaction of CD40 and CD40L increases STAT3 activation and the proliferation of MDA-MD231 cells. (A) MDA-MB231 cells and activated T cells were directly co-cultured at the ratio of 1:5 for 24 hrs in the presence of anti-IL-17 neutralizing antibody or anti-CD40 neutralizing antibody (2 μg/ml/each) on 96-well plate, and then cells were cultured for 24 hrs. After the addition of 1 μCi/mL of [^3^H]-thymidine, cells were culture for another 18 hrs. And the proliferation of cells was measured as described in *Materials and Methods*. Data represents mean ± S.D. The assay was performed in quadruplicate and result is the representative of three independent experiments. (B) MDA-MB231 cells were cultured in the presence of recombinant IL-17 (rIL-17, 50 ng/ml) for 15, 30 and 60 min. In addition, cells were cultured with direct co-culture supernatant of MDA-MB231 cells and activated T cells in the presence or absence of anti-IL-17 neutralizing antibody (nAb). And then, the activation of STAT3 was examined by western blot as described in *Materials and Methods*. Lane 1: Control, Lane 2: rIL-17 (50 ng/ml), Lane 3: Direct co-culture supernatant of MDA-MB231 cells and activated T cells, Lane 4: Direct co-culture supernatant of MDA-MB231 cells and activated T cells with IL-17 nAb, Lane 5: Indirect co-culture supernatant of MDA-MB231 cells and activated T cells. β-actin was used as a loading control. Result is the representative of three independent experiments. (C) MDA-MB231 cells and activated T cells were directly co-cultured at the ratio of 1:5 for 24 hrs in the presence of 20 μM of AG490 (STAT3 inhibitor) or anti-IL-17 nAb (2 μg/ml) on 96-well plate, and then cells were cultured for 24 hrs. After the addition of 1 μCi/mL of [^3^H]-thymidine, cells were culture for another 18 hrs. Direct co-culture supernatant of CD40-non expressing breast cancer cell line, Hs578T and activated T cells was used as a negative control. The proliferation of cells was measured as described in *Materials and Methods*. Data represents mean ± S.D. The assay was performed in quadruplicate and result is the representative of three independent experiments.

Tumor growth is suppressed in IL-17 knockout mice and IL-17/IFN-γ double-knockout mice [38], and a mechanism for this knockout is that IL-17 induces the production of IL-6 by tumor cells, which in turn promotes tumor growth through a STAT3–dependent pathway. Because Th17 cells were increased by the CD40-CD40L interaction between the MDA-MB231 cells and activated T cells, the proliferation of MDA-MB231 cells was examined next. MDA-MB231 cell proliferation was highly increased when MDA-MB231 cells were incubated with the direct co-culture supernatant but not with the indirect co-culture supernatant from the MDA-MB231 cells and activated T cells. However, this increase in proliferation was attenuated by neutralizing IL-17 with anti-IL-17 antibody in the direct co-culture supernatant (Fig 5A). It was also attenuated by incubating the cells with supernatant obtained from the direct co-culture of activated T cells and MDA-MB231 cells pre-treated with anti-CD40 neutralizing antibodies. Because IL-17 can promote tumor growth through the STAT3 signaling pathway [38], we investigated whether STAT3 is involved in IL-17-mediated MDA-MB231 cell proliferation. When MDA-MB231 cells were treated with recombinant IL-17 (rIL-17), phosphorylated STAT3 increased up until 30 min after treatment and then decreased at 60 min to the same extent as the MDA-MB231 cells that were incubated with the direct co-culture supernatant of the MDA-MB231 cells and activated T cells (Fig 5B). However, the activation of STAT3 was inhibited when the MDA-MB231 cells were incubated with direct co-culture supernatant pre-treated with anti-IL-17 neutralizing antibody. Moreover, the increased proliferation of the MDA-MB231 cells by the direct co-culture supernatant was attenuated by treatment with AG490, a STAT3 inhibitor (Fig 5C). In addition, the direct co-culture supernatant of Hs578T cells and activated T cells did not induce the proliferation of MDA-MB231 cells.

## Discussion

CD40 is mainly expressed on antigen presenting cells (APCs) including B cells, dendritic cells, and macrophages to activate immune responses after interaction with its ligand, CD40L, on activated T cells [39–41]. There are recent reports regarding CD40 expression on several kinds of tumor cells [42–45]; however, its role in tumor cells is not clearly known.

We found that CD40 is highly expressed in the breast cancer cell line MDA-MB231 and hardly expressed in the breast cancer cell line Hs578T (Fig 1). However, CD40L is not expressed in both of the cell lines (S2 Fig). This means that the possibility of an interaction between CD40 and CD40L in MDA-MB231 cells could be ignored. In contrast, CD40L is expressed in activated T cells (Fig 2A); however, CD40 is not expressed in activated T cells (S3 Fig). Therefore, an interaction between CD40 and CD40L in activated T cells could also be ignored. Based on our findings regarding CD40 and CD40L expression, the function of CD40 signaling in breast cancer cells was investigated with CD40 stimulating antibody, soluble CD40L and activated T cells.

First, we investigated the role of CD40 in the proliferation of MDA-MB231 cells because its original function in the immune system is that of a key mediator for the proliferation and differentiation of B cells [46, 47]. In contrast to our expectation, the proliferation of MDA-MB231 cells was not observed by stimulation of CD40 with anti-CD40 antibody (Fig 1B). There are several molecules that have different functions according to their origins [48, 49]. For example, IL-18 produced by immune cells has an anti-tumor activity; however, IL-18 produced by tumor cells has an important role in the immune escape process [50, 51]. Tumor cells secret TGF-β and IL-10, which are the most well-known immunosuppressive cytokines, to escape immune surveillance [30, 52]. For this reason, we next examined whether CD40 also has a role in the immune escape mechanism of breast cancer through the production of TGF-β. As shown in Fig 1C and 1D, CD40 stimulation increased the expression of TGF-β mRNA transcripts in MDA-MB231 cells. In addition, its secretion from MDA-MB231 cells is also increased by CD40 stimulation when treating the cells with its agonistic antibody and soluble ligand and by co-culturing with CD40L-expressing activated T cells (Fig 1E and 1F). Moreover, the increase in TGF-β production is completely blocked by pre-treatment with CD40 and CD40L neutralizing antibodies or by indirect co-culture of MDA-MB231 cells and activated T cells (Fig 2C). Therefore, our data not only show that CD40 has a role in the production of TGF-β from the tumor to suppress the immune system in the tumor microenvironment, but also has a novel mechanism in the increase of TGF-β production by CD40 stimulation.

Immune suppression by TGF-β is achieved by regulating cells in adaptive immunity components, such as T and B cells, as well as in innate immunity components, such as natural killer (NK) cells [42, 53, 54]. Additionally, it was recently reported that TGF-β suppresses immune responses by promoting regulatory T cell (Treg) induction and Th17 differentiation from conventional T cells [43, 44]. Because forkhead box protein 3 (Foxp3) is expressed when T cells become Treg, we examined whether Foxp3 is increased in T cells after stimulation of CD40L with anti-CD40L agonistic Ab or by co-culture with CD40-expressing MDA-MB231 cells. However, we could not detect an increase in Foxp3 expression (data not shown). Therefore, we next examined the role of the CD40-CD40L interaction in Th17 differentiation.

As shown in Fig 1E and 1F, MDA-MB231 cells produced TGF-β by stimulating CD40 with anti-CD40 agonistic antibody or sCD40L, and by co-culture with activated T cells. TGF-β has multiple cellular responses including the induction of cell growth inhibition, differentiation, wound healing and apoptosis [45]. TGF-β signaling can act as a tumor suppressor or tumor promoter depending on the tumor type and the stage of tumor progression [55]. Furthermore, TGF-β has a crucial role in Th17 cell lineage commitment [29]. It is known that TGF-β and either IL-6 or IL-21 are essential factors in the induction of Th17 differentiation [43]. Furthermore, we showed that the production of IL-1β, IL-6 and IL-21 is increased by direct co-culture of MBA-MB231 cells with activated T cells (Fig 3B–3D). This result suggests that the optimal condition for Th17 differentiation could be induced by the interaction between CD40 on the surface of the MDA-MB231 cells and CD40L on the surface of the activated T cells. In fact, we observed an increased population of IL-17-producing CD4^+^ T cells (Fig 3A). Our study coincides with a report that CD40-CD40L cross-talk is important in Th17 development [11].

As seen in Fig 1B, there was no effect from CD40 stimulation on the proliferation of MDA-MB231 cells *in vitro*. The proliferation of MDA-MB231 cells did not change even though TGF-β production was increased by CD40 stimulation. However, we found that the production and secretion of IL-17 were increased through the CD40-CD40L interaction. It is still controversial whether IL-17 has a tumor-suppressing effect or tumor-promoting effect [56]. In our study, IL-17 increased the proliferation of MDA-MB231 cells through the activation of STAT3. IL-17-mediated proliferation of MDA-MB231 cells was inhibited by the treatment with a STAT3 inhibitor AG490 and anti-IL-17 neutralizing antibody (Fig 5). CD40L is expressed in many cells including mast cells, macrophages, basophils, NK cells, B cells, smooth muscle cells, endothelial cells, and epithelial cells [44]. Based on the activated T cells, it seems that these cells also get a signal through CD40L found on the surface of the cells by interacting with CD40 on the surface of the MDA-MB231 cells. CD40 has an important role in generating T cell responses against viruses and bacteria through the interaction with CD40L on T cells [57, 58]. In particular, the role of CD40 in generating T cell responses provides the possibility of eliciting effective anti-tumor immune responses because CD40 on APC can deliver co-stimulatory signaling for the activation of CD8^+^ cells directly without the activation of CD4^+^ helper T cells [59, 60]. In fact, it was reported that efficient cytotoxic T lymphocytes responded against tumors when administering CD4 knock-out mice with CD40 activating monoclonal antibody [61]. That is, the ligation of CD40 on B cells up-regulates their co-stimulatory activity, and these cells might have a role in the generation of cytotoxic T lymphocyte responses against tumors. However, the cytotoxicity of activated T cells against MDA-MB231 cells was not observed because unlike B cells, MDA-MB231 cells do not express co-stimulatory molecules on their surface. Therefore, the significance of activated T cells delivering their signals through CD40L will be further investigated. In addition, *in vivo* administration of CD40 stimulating antibody for the induction of cytotoxic T lymphocyte responses against tumors should be considered carefully because it can suppress immune responses through an increase in TGF-β production from tumors based on the results of this study.

In summary, an interaction between CD40 on MDA-MB231 breast cancer cells and CD40L on activated T cells increases the production of Th17 differentiating cytokines including TGF-β, IL-1β, IL-6, and IL-21. Moreover, the increased IL-17 promotes the proliferation of the breast cancer line MDA-MB231 through the activation of STAT3. Therefore, it seems that CD40 on tumor cells is closely related to the immune escape of tumors. Thus, by modulating the interaction between CD40 on tumor cells and CD40L on activated T cells, the efficiency of cancer immunotherapy could be increased by regulating the TGF-β production in a tumor microenvironment.

## Supporting Information

S1 Fig CD40 expression is down-regulated on MDA-MB231 cells by CD40 siRNA transfection. MDA-MB231 cells (1.5 x 10^5^/well) were seeded on 6-well plate and then transfected with 20 nM of CD40 siRNA and control siRNA in the mixture of serum free media and oligofectamine as described in *Materials and Methods*. After 72 hrs, the down-regulation of CD40 expression on MDA-MB231 cells by CD40 siRNA transfection was confirmed by flow cytometry analysis. Result is the representative of three independent experiments.
(TIF)

S2 Fig CD40L is not expressed on MDA-MB231 and Hs578T cells. Both breast cancer cell lines, MDA-MB231 and Hs578T were collected at continuous log phase of growth. The expression of CD40L was examine by staining with PE-conjugated anti-human CD40L antibody (1 μg/ml), as described in *Materials and Methods*. Result is the representative of three independent experiments.
(TIF)

S3 Fig CD40 expression is not induced on activated T cells. Human T cells (2.5x10^6^/ml) purified from human PBMCs were activated as described in *Materials and Methods*. And then CD40 expression on activated T cells was examine by staining with PE-conjugated anti-human CD40 antibody (1 μg/ml). Result is the representative of three independent experiments.
(TIF)

## References

[1] van Kooten C, Banchereau J. CD40-CD40 ligand. J Leukoc Biol. 2000;67(1):2–17. 10647992 10.1002/jlb.67.1.2

[2] Eliopoulos AG, Davies C, Knox PG, Gallagher NJ, Afford SC, Adams DH, et al CD40 induces apoptosis in carcinoma cells through activation of cytotoxic ligands of the tumor necrosis factor superfamily. Mol Cell Biol. 2000;20(15):5503–15. 10891490 10.1128/mcb.20.15.5503-5515.2000 PMC86001

[3] McDyer JF, Goletz TJ, Thomas E, June CH, Seder RA. CD40 ligand/CD40 stimulation regulates the production of IFN-γ from human peripheral blood mononuclear cells in an IL-12-and/or CD28-dependent manner. The Journal of Immunology. 1998;160(4):1701–7. 9469427

[4] Qian Y, Hamrah P, Boisgerault F, Yamagami S, Vora S, Benichou G, et al Mechanisms of immunotherapeutic intervention by anti-CD154 (CD40L) antibody in high-risk corneal transplantation. J Interferon Cytokine Res. 2002;22(12):1217–25. 12581495 10.1089/10799900260475740

[5] Takatsu K. [Role of interleukin-5 in immune regulation and inflammation]. Nihon Rinsho. 2004;62(10):1941–51. 15500144

[6] Pasch MC, Timár KK, van Meurs M, Heydendael VM, Bos JD, Laman JD, et al In situ demonstration of CD40—and CD154—positive cells in psoriatic lesions and keratinocyte production of chemokines by CD40 ligation in vitro. The Journal of pathology. 2004;203(3):839–48. 15221944 10.1002/path.1581

[7] Johnson-Léger C, Christenson JR, Holman M, Klaus GG. Evidence for a critical role for IL-2 in CD40-mediated activation of naive B cells by primary CD4 T cells. The Journal of Immunology. 1998;161(9):4618–26. 9794390

[8] Takatsu K. Cytokines involved in B-cell differentiation and their sites of action. Exp Biol Med. 1997;215(2):121–33. 9160040 10.3181/00379727-215-44119

[9] Bishop GA, Hostager BS. Signaling by CD40 and its mimics in B cell activation. Immunol Res. 2001;24(2):97–109. 11594459 10.1385/IR:24:2:097

[10] Wingett DG, Vestal RE, Forcier K, Hadjokas N, Nielson CP. CD40 is functionally expressed on human breast carcinomas: variable inducibility by cytokines and enhancement of Fas-mediated apoptosis. Breast Cancer Res Treat. 1998;50(1):27–36. 9802617 10.1023/a:1006012607452

[11] Iezzi G, Sonderegger I, Ampenberger F, Schmitz N, Marsland BJ, Kopf M. CD40–CD40L cross-talk integrates strong antigenic signals and microbial stimuli to induce development of IL-17-producing CD4+ T cells. Proceedings of the National Academy of Sciences. 2009;106(3):876–81. 10.1073/pnas.0810769106 19136631 PMC2630101

[12] Batrla R, Linnebacher M, Rudy W, Stumm S, Wallwiener D, Gückel B. CD40-expressing carcinoma cells induce down-regulation of CD40 ligand (CD154) and impair T-cell functions. Cancer Res. 2002;62(7):2052–7. 11929824

[13] Jakowlew SB. Transforming growth factor-β in cancer and metastasis. Cancer Metastasis Rev. 2006;25(3):435–57. 16951986 10.1007/s10555-006-9006-2

[14] Porst M, Daniel C, Plank C, Schocklmann HO, Reinhardt DP, Hartner A. Induction and coexpression of latent transforming growth factor β-binding protein-1 and fibrillin-1 in experimental glomerulonephritis. Nephron Experimental Nephrology. 2005;102(3–4):e99–e104. 16282705 10.1159/000089688

[15] Horimoto M, Kato J, Takimoto R, Terui T, Mogi Y, Niitsu Y. Identification of a transforming growth factor beta-1 activator derived from a human gastric cancer cell line. Br J Cancer. 1995;72(3):676 7669580 10.1038/bjc.1995.393 PMC2033878

[16] Abe M, Oda N, Sato Y, Shibata K, Yamasaki M. Augmented binding and activation of latent transforming growth factor-β by a tryptic fragment of latency associated peptide. Endothelium. 2002;9(1):25–36. 12901358 10.1080/10623320210710

[17] Munger JS, Harpel JG, Gleizes P-E, Mazzieri R, Nunes I, Rifkin DB. Latent transforming growth factor-bold beta: Structural features and mechanisms of activation. Kidney Int. 1997;51:1376–82. 9150447 10.1038/ki.1997.188

[18] Annes JP, Chen Y, Munger JS, Rifkin DB. Integrin αvβ6-mediated activation of latent TGF-β requires the latent TGF-β binding protein-1. The Journal of cell biology. 2004;165(5):723–34. 15184403 10.1083/jcb.200312172 PMC2172370

[19] Imamura T, Hikita A, Inoue Y. The roles of TGF-β signaling in carcinogenesis and breast cancer metastasis. Breast Cancer. 2012;19(2):118–24. 10.1007/s12282-011-0321-2 22139728

[20] Elliott RL, Blobe GC. Role of transforming growth factor Beta in human cancer. J Clin Oncol. 2005;23(9):2078–93. 15774796 10.1200/JCO.2005.02.047

[21] Moses H, Barcellos-Hoff MH. TGF-β biology in mammary development and breast cancer. Cold Spring Harb Perspect Biol. 2011;3(1):a003277 10.1101/cshperspect.a003277 20810549 PMC3003461

[22] Katsuno Y, Hanyu A, Kanda H, Ishikawa Y, Akiyama F, Iwase T, et al Bone morphogenetic protein signaling enhances invasion and bone metastasis of breast cancer cells through Smad pathway. Oncogene. 2008;27(49):6322–33. 10.1038/onc.2008.232 18663362

[23] Kang Y, Siegel PM, Shu W, Drobnjak M, Kakonen SM, Cordón-Cardo C, et al A multigenic program mediating breast cancer metastasis to bone. Cancer Cell. 2003;3(6):537–49. 12842083 10.1016/s1535-6108(03)00132-6

[24] Shin H, Lim CK, Choi IY, Lee DY, Noh DY, Ryu MH, et al Study of plasma transforming growth factor-β 1 level as a useful tumor marker in various cancers. Immune Netw. 2001;1(2):143–50.

[25] Lim CK, Shin H, Choi IY, Chung BH, Ryu MH, Bang YJ, et al Study of plasma TGF-β1 level as a useful tumor marker in gastric cancer and prostate cancer. Immune Netw. 2001;1(3):260–5.

[26] Randolph DA, Fathman CG. Cd4+ Cd25+ regulatory T cells and their therapeutic potential. Annu Rev Med. 2006;57:381–402. 16409156 10.1146/annurev.med.57.121304.131337

[27] McHugh RS, Whitters MJ, Piccirillo CA, Young DA, Shevach EM, Collins M, et al CD4^+^CD25^+^ Immunoregulatory T Cells: Gene Expression Analysis Reveals a Functional Role for the Glucocorticoid-Induced TNF Receptor. Immunity. 2002;16(2):311–23. 11869690 10.1016/s1074-7613(02)00280-7

[28] Lee YK, Mukasa R, Hatton RD, Weaver CT. Developmental plasticity of Th17 and Treg cells. Curr Opin Immunol. 2009;21(3):274–80. 10.1016/j.coi.2009.05.021 19524429

[29] Mangan PR, Harrington LE, O'Quinn DB, Helms WS, Bullard DC, Elson CO, et al Transforming growth factor-β induces development of the TH17 lineage. Nature. 2006;441(7090):231–4. 16648837 10.1038/nature04754

[30] Park H-Y, Wakefield LM, Mamura M. Regulation of tumor immune surveillance and tumor immune subversion by TGF-β. Immune Netw. 2009;9(4):122–6. 10.4110/in.2009.9.4.122 20157598 PMC2816944

[31] Petersen C, Christensen E, Andresen B, Møller B. Internalization, lysosomal degradation and new synthesis of surface membrane CD4 in phorbol ester-activated T-lymphocytes and U-937 cells. Exp Cell Res. 1992;201(1):160 1612121 10.1016/0014-4827(92)90360-k

[32] Baran J, Kowalczyk D, Oz M, Zembala M. Three-color flow cytometry detection of intracellular cytokines in peripheral blood mononuclear cells: comparative analysis of phorbol myristate acetate-ionomycin and phytohemagglutinin stimulation. Clin Diagn Lab Immunol. 2001;8(2):303–13. 11238213 10.1128/CDLI.8.2.303-313.2001 PMC96054

[33] Spriggs M, Armitage R, Strockbine L, Clifford K, Macduff B, Sato T, et al Recombinant human CD40 ligand stimulates B cell proliferation and immunoglobulin E secretion. The Journal of experimental medicine. 1992;176(6):1543–50. 1281209 10.1084/jem.176.6.1543 PMC2119450

[34] Kim R, Emi M, Tanabe K. Cancer immunosuppression and autoimmune disease: beyond immunosuppressive networks for tumour immunity. Immunology. 2006;119(2):254–64. 17005005 10.1111/j.1365-2567.2006.02430.x PMC1782355

[35] Hatton RD. TGF-β in Th17 cell development: the truth is out there. Immunity. 2011;34(3):288–90. 10.1016/j.immuni.2011.03.009 21435582 PMC3097895

[36] Awasthi A, Kuchroo VK. Th17 cells: from precursors to players in inflammation and infection. Int Immunol. 2009:dxp021 10.1093/intimm/dxp021 19261692 PMC2675030

[37] Ivanov II, McKenzie BS, Zhou L, Tadokoro CE, Lepelley A, Lafaille JJ, et al The orphan nuclear receptor RORγt directs the differentiation program of proinflammatory IL-17+ T helper cells. Cell. 2006;126(6):1121–33. 16990136 10.1016/j.cell.2006.07.035

[38] Wang L, Yi T, Kortylewski M, Pardoll DM, Zeng D, Yu H. IL-17 can promote tumor growth through an IL-6–Stat3 signaling pathway. The Journal of experimental medicine. 2009;206(7):1457–64. 10.1084/jem.20090207 19564351 PMC2715087

[39] Néron S, Racine C, Roy A, Guérin M. Differential responses of human B-lymphocyte subpopulations to graded levels of CD40–CD154 interaction. Immunology. 2005;116(4):454–63. 16313359 10.1111/j.1365-2567.2005.02244.x PMC1802436

[40] Watanabe S, Kagamu H, Yoshizawa H, Fujita N, Tanaka H, Tanaka J, et al The duration of signaling through CD40 directs biological ability of dendritic cells to induce antitumor immunity. The Journal of Immunology. 2003;171(11):5828–36. 14634092 10.4049/jimmunol.171.11.5828

[41] Maruo S, Oh-hora M, Ahn H-J, Ono S, Wysocka M, Kaneko Y, et al B cells regulate CD40 ligand-induced IL-12 production in antigen-presenting cells (APC) during T cell/APC interactions. The Journal of Immunology. 1997;158(1):120–6. 8977182

[42] Laouar Y, Sutterwala FS, Gorelik L, Flavell RA. Transforming growth factor-β controls T helper type 1 cell development through regulation of natural killer cell interferon-γ. Nature immunology. 2005;6(6):600–7. 15852008 10.1038/ni1197

[43] Wan YY, Flavell RA. ‘Yin-Yang’functions of transforming growth factor‐β and T regulatory cells in immune regulation. Immunological reviews. 2007;220(1):199–213. 17979848 10.1111/j.1600-065X.2007.00565.x PMC2614905

[44] Park S-H, Cho G, Park S-G. NF-κB Activation in T Helper 17 Cell Differentiation. Immune network. 2014;14(1):14–20. 10.4110/in.2014.14.1.14 24605076 PMC3942503

[45] Kim IY, Kim MM, Kim S. Transforming growth factor-beta: biology and clinical relevance. Journal of biochemistry and molecular biology. 2005;38(1):1 15715939 10.5483/bmbrep.2005.38.1.001

[46] Fecteau JF, Néron S. CD40 stimulation of human peripheral B lymphocytes: distinct response from naive and memory cells. The Journal of Immunology. 2003;171(9):4621–9. 14568936 10.4049/jimmunol.171.9.4621

[47] Park J-Y, Yoon SH, Kim E-K, Yun S-O, Sohn H-J, Kim T-G. Enhancement of Proliferation and Antigen Presentation of Human B Cells in Vitro by K562 Cells Expressing CD40L. Immune Netw. 2007;7(2):80–6.

[48] Reddy P. Interleukin-18: recent advances. Curr Opin Hematol. 2004;11(6):405–10. 15548995 10.1097/01.moh.0000141926.95319.42

[49] Mühl H, Pfeilschifter J. Interleukin-18 bioactivity: a novel target for immunopharmacological anti-inflammatory intervention. Eur J Pharmacol. 2004;500(1):63–71. 15464021 10.1016/j.ejphar.2004.07.012

[50] Carbone A, Rodeck U, Mauri FA, Sozzi M, Gaspari F, Smirne C, et al Research Paper Human Pancreatic Carcinoma Cells Secrete Bioactive Interleukin-18 after Treatment with 5-Fluorouracil. Cancer Biol Ther. 2005;4(2):231–41. 15684607 10.4161/cbt.4.2.1476

[51] Cho D, Song H, Kim YM, Houh D, Hur DY, Park H, et al Endogenous interleukin-18 modulates immune escape of murine melanoma cells by regulating the expression of Fas ligand and reactive oxygen intermediates. Cancer Res. 2000;60(10):2703–9. 10825144

[52] PRIES R, THIEL A, BROCKS C, WOLLENBERG B. Secretion of tumor-promoting and immune suppressive cytokines by cell lines of head and neck squamous cell carcinoma. In Vivo. 2006;20(1):45–8. 16433027

[53] Cazac BB, Roes J. TGF-β receptor controls B cell responsiveness and induction of IgA in vivo. Immunity. 2000;13(4):443–51. 11070163 10.1016/s1074-7613(00)00044-3

[54] Gorelik L, Flavell RA. Abrogation of TGFβ signaling in T cells leads to spontaneous T cell differentiation and autoimmune disease. Immunity. 2000;12(2):171–81. 10714683 10.1016/s1074-7613(00)80170-3

[55] Padua D, Massagué J. Roles of TGFβ in metastasis. Cell Res. 2008;19(1):89–102. 10.1038/cr.2008.316 19050696

[56] Martin F, Apetoh L, Ghiringhelli F. Controversies on the role of Th17 in cancer: a TGF-β-dependent immunosuppressive activity? Trends Mol Med. 2012;18(12):742–9. 10.1016/j.molmed.2012.09.007 23083809

[57] Sarawar SR, Lee BJ, Reiter SK, Schoenberger SP. Stimulation via CD40 can substitute for CD4 T cell function in preventing reactivation of a latent herpesvirus. Proceedings of the National Academy of Sciences. 2001;98(11):6325–9. 11353832 10.1073/pnas.101136898 PMC33467

[58] Cotter RL, Zheng J, Che M, Niemann D, Liu Y, He J, et al Regulation of human immunodeficiency virus type 1 infection, β-chemokine production, and CCR5 expression in CD40L-stimulated macrophages: immune control of viral entry. J Virol. 2001;75(9):4308–20. 11287580 10.1128/JVI.75.9.4308-4320.2001 PMC114176

[59] Schoenberger SP, Toes RE, van der Voort EI, Offringa R, Melief CJ. T-cell help for cytotoxic T lymphocytes is mediated by CD40–CD40L interactions. Nature. 1998;393(6684):480–3. 9624005 10.1038/31002

[60] Szomolanyi-Tsuda E, Brien JD, Dorgan JE, Welsh RM, Garcea RL. The role of CD40-CD154 interaction in antiviral T cell-independent IgG responses. The Journal of Immunology. 2000;164(11):5877–82. 10820268 10.4049/jimmunol.164.11.5877

[61] van Mierlo GJ, den Boer AT, Medema JP, van der Voort EI, Fransen MF, Offringa R, et al CD40 stimulation leads to effective therapy of CD40− tumors through induction of strong systemic cytotoxic T lymphocyte immunity. Proceedings of the National Academy of Sciences. 2002;99(8):5561–6. 11929985 10.1073/pnas.082107699 PMC122809

