# Supplementary figures and images for: Direct Interaction of CD40 on Tumor Cells with CD40L on T Cells Increases the Proliferation of Tumor Cells by Enhancing TGF-β Production and Th17 Differentiation
Source: PLoS One. 2015 May 18;10(5):e0125742. doi: 10.1371/journal.pone.0125742 (PMC4436336; doi:10.1371/journal.pone.0125742)

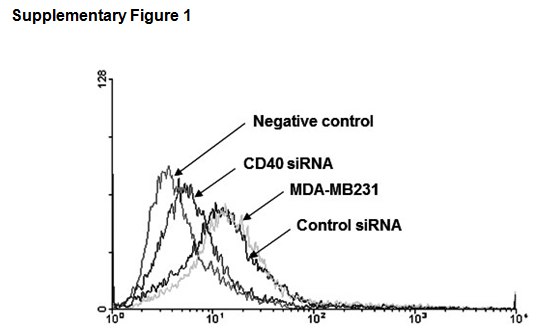

Supplement: S1 Fig — MDA-MB231 cells (1.5 x 10^5^/well) were seeded on 6-well plate and then transfected with 20 nM of CD40 siRNA and control siRNA in the mixture of serum free media and oligofectamine as described in Materials and Methods. After 72 hrs, the down-regulation of CD40 expression on MDA-MB231 cells by CD40 siRNA transfection was confirmed by flow cytometry analysis. Result is the representative of three independent experiments. (TIF) [file pone.0125742.s001.tif]

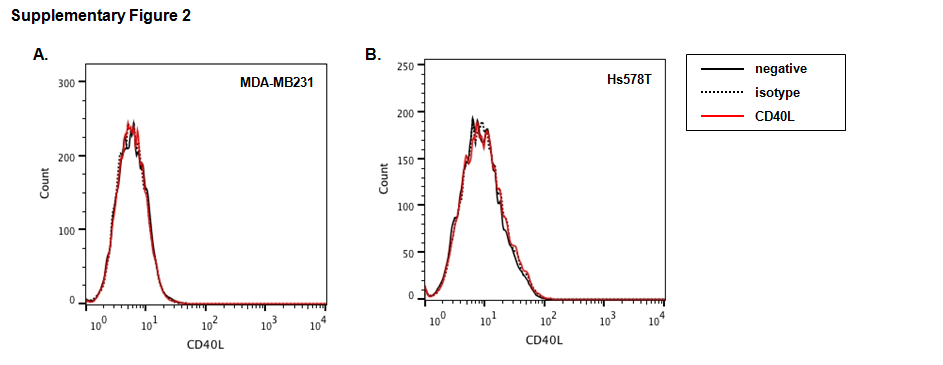

Supplement: S2 Fig — Both breast cancer cell lines, MDA-MB231 and Hs578T were collected at continuous log phase of growth. The expression of CD40L was examine by staining with PE-conjugated anti-human CD40L antibody (1 μg/ml), as described in Materials and Methods. Result is the representative of three independent experiments. (TIF) [file pone.0125742.s002.tif]

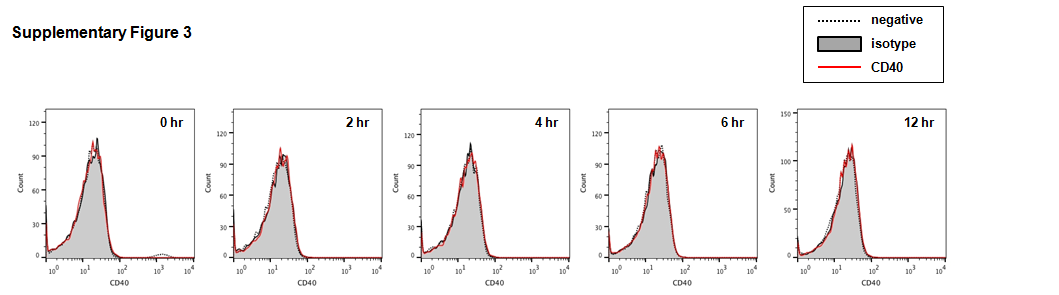

Supplement: S3 Fig — Human T cells (2.5x10^6^/ml) purified from human PBMCs were activated as described in Materials and Methods. And then CD40 expression on activated T cells was examine by staining with PE-conjugated anti-human CD40 antibody (1 μg/ml). Result is the representative of three independent experiments. (TIF) [file pone.0125742.s003.tif]
